# Trends and correlates of meeting 24-hour movement guidelines: a 15-year study among 167,577 Thai adults

**DOI:** 10.1186/s12966-020-01011-9

**Published:** 2020-08-24

**Authors:** Nucharapon Liangruenrom, Dorothea Dumuid, Melinda Craike, Stuart J. H. Biddle, Zeljko Pedisic

**Affiliations:** 1 Institute for Health and Sport, Victoria University, PO Box 14428, Melbourne, VIC 8001 Australia; 2 Institute for Population and Social Research, Mahidol University, Phutthamonthon Sai 4 Road, Salaya, Phutthamonthon, Nakhon Pathom, 73170 Thailand; 3 Allied Health and Human Performance, Alliance for Research in Exercise, Nutrition and Activity, University of South Australia, Frome Road, Adelaide, South Australia 5001 Australia; 4 Mitchell Institute for Education and Health Policy, Victoria University, PO Box 14428, Melbourne, VIC 8001 Australia; 5 Centre for Health Research, University of Southern Queensland, Education City, 37 Sinnathamby Boulevard, Springfield Central, Queensland 4300 Australia

**Keywords:** Time-use data, Physical activity, Sedentary behaviour, Sleep, ICATUS, Time-use epidemiology

## Abstract

**Background:**

Time spent in physical activity (PA), sedentary behaviour (SB), and sleep always takes up the whole day. New public health guidelines combining recommendations for PA, SB, and sleep have been issued in several countries. Thailand was the first country to release the 24-h guidelines for adults. Currently, there is no evidence on the population prevalence of meeting 24-h movement guidelines in Thailand. This study, therefore, aimed to determine 15-year trends and associations of meeting 24-h movement guidelines among Thai adults.

**Method:**

We analysed cross-sectional data from 2001, 2004, 2009, and 2015 Thai Time-Use Surveys, coded using the International Classification of Activities for Time-Use Statistics (ICATUS). All ICATUS-based activities were categorised into moderate-to-vigorous PA (MVPA), light PA (LPA), SB, and sleep based on a previously developed classification system. A total of 167,577 adult participants were included. The participants were classified according to the Thai 24-h movement guidelines into meeting or not meeting the following criteria: 1) ≥150 min/week of MVPA; 2) interrupting SB every 2 h; 3) sleeping 7–9 h per day; and 4) adhering to all three guidelines.

**Results:**

In 2015, the prevalence of adults who met the MVPA, SB, sleep, and overall recommendations was 81.7, 44.6, 56.4, and 21.3%, respectively. A significant linear increase was found for the prevalence of meeting the SB recommendation, while the prevalence meeting the MVPA, sleep, and overall recommendations was lowest in 2001, peaked in 2004 or 2009, and declined in 2015. The lowest odds for meeting the 24-h guidelines were found among males, those living in urban areas, inhabitants of Bangkok and South Thailand, unemployed, and those with low education level.

**Conclusions:**

Despite promising trends in the prevalence of meeting PA, SB, and sleep recommendations, a majority of Thai adults still do not meet the overall 24-h movement guidelines. Further actions are needed to promote more MVPA, less SB, and adequate sleep in Thai adults, particularly among males, those living in urban areas, inhabitants of Bangkok and South Thailand, unemployed, and those with low education level.

## Background

Given that everyone has a fixed 24-h budget in a day and the time spent in physical activity (PA), sedentary behaviour (SB), and sleep always takes up the whole day, any change in the time spent in one of these behaviours necessarily affects the remaining behaviours [1–4]. Recent epidemiological research has therefore often considered these behaviours collectively in relation to health. Growing evidence shows that high PA, low sedentary time, and adequate sleep duration are collectively associated with a range of health benefits, such as lower body mass index (BMI), low waist circumference, and high aerobic fitness [5–10]. Based on the emerging evidence and a better understanding of the importance of considering these behaviours holistically, new public health guidelines that combine recommendations for PA, SB, and sleep have been issued in several countries [11–19]. Canada pioneered the development of such guidelines, and in 2016 they launched the first national 24-h movement guidelines [16]. Soon after, following the Canadian example, Australia, Finland, New Zealand, South Africa, and Thailand issued their 24-h movement guidelines [11–15]. To date, a few additional countries, such as Croatia and the United Kingdom, have made initial steps in the process of adopting similar public health guidelines [17, 18]. The World Health Organization (WHO) supported the integrative approach to movement behaviours and has recently released 24-h guidelines for children under 5 years of age [19].

Time-use surveys provide comprehensive data on common activities that the general population performs in a day [20]. Since the 1960s, time-use data have been collected worldwide in over 85 countries [20]. With contextualised information across life domains and international availability, these surveys have been a valuable source of data for epidemiological research on movement behaviours [21]. They are considered to provide sufficiently valid and reliable PA, SB, and sleep estimates for observational studies [3, 21–26].

To date, time-use data have been used to examine population prevalence and correlates of PA and SB, particularly in high-income countries [23, 27–37]. Some studies also used time-use survey data to explore the prevalence of PA/SB or meeting PA recommendations and trends over time [38–41]. Assessing the prevalence of meeting the 24-h movement guidelines has started to gain momentum in national health monitoring studies [42], and several studies have examined associations of meeting the guidelines with health and psychological wellbeing among youth [43–45]. The 24-h movement guidelines are a new benchmark for assessing healthy use of time in the population. Determining the prevalence of meeting these new public health guidelines is essential to inform future health promotion and disease prevention policies and interventions. However, only a few previous studies have assessed the prevalence and correlates of meeting the 24-h movement guidelines in national-representative samples of adults [42, 43, 45]. Further, no studies have employed data from time-use surveys for this purpose.

Thailand is an upper-middle income country, and its Government has a progressive approach for improving population health. For example, universal health coverage was introduced in 2002, and significant progress was made in the primary prevention of chronic diseases, particularly by taking actions to promote PA in the population [46, 47]. Thailand recently released the 1st National Strategic Plan for the Promotion of Physical Activity (2018–2030), and its Ministry of Public Health took part in issuing the Bangkok Declaration on Physical Activity for Global Health and Sustainable Development [48, 49]. The Thai National Strategic Plan for the Promotion of Physical Activity was primarily based on the Global Recommendations on Physical Activity for Health, developed by the WHO [13, 50]. However, taking into account emerging evidence on collective health impacts of PA, SB, and sleep, Thailand issued holistic, national 24-h movement guidelines for five different population groups in 2017 [13]. These target population groups include: 1) pregnant and postpartum women; 2) early years (0–5 years); 3) school-aged children and adolescents (6–17 years); 4) adults (18–59 years); and 5) older adults (60 years and older). To our knowledge, Thailand was the first country to issue 24-h movement guidelines, including PA, SB, and sleep recommendations, for adults, older adults, and pregnant and postpartum women. This is consistent with the progressive thinking and strong commitment of the Thai Government to improve national preventative healthcare.

Currently, there is no evidence on the population prevalence of meeting 24-h movement guidelines in Thailand. There is also a lack of evidence on PA and SB trends in the Thai population [51]. In addition, the authors are not aware of any studies that have examined the temporal changes of factors associated with meeting the integrated movement guidelines. This study, therefore, aimed to determine 15-year trends of meeting 24-h movement guidelines and to examine how associations between sociodemographic variables and meeting these recommendations have changed over time.

Previous research from other countries has shown that, in the last few decades, in some countries, there has been a clear shift from physically active to sedentary lifestyles [39], while sleep duration in adult populations has generally remained constant [52–54]. We, therefore, hypothesised that among Thai adults PA will have decreased, SB increased, and sleep duration remained stable since 2001.

## Methods

### Thai national time-use survey

The National Statistical Office (NSO) conducted cross-sectional Thai National Time-Use Surveys in 2001, 2004, 2009, and 2015 [55–58]. The NSO randomly selected private households by using the three-stage stratified sampling method [55–58]. The stratification was according to five Thai regions (Bangkok, Central, North, North-East, and South), 77 provinces, and municipal and non-municipal areas called *Enumerations* (EAs) [55–58]. The Municipality Act, B.E. 2496 (1953) divides household areas into municipal or urban areas, where a single administrative district has powers of self-government (also known as cities), and non-municipal or rural areas, such as villages [59]. A random sample of EAs was selected within each province. A random sample of households was selected from a list of dwellings within each selected EA. One member from each of the selected households was randomly selected. Among the selected household members, data were collected using a combination of a direct interview and self-administered questionnaires. The participants were asked to report all activities they engaged in on a selected day, including the start and end time for each activity. The NSO trained officers recorded and coded participants’ daily activities over a 24-h day (from 0:00 to 24:00 h) using the ICATUS coding rules and a computer-assisted coding device [60, 61]. The reported activities were coded for 10-min intervals, except in the survey year 2001, when the reported activities were recorded and coded with no fixed time intervals. The surveys captured main (primary) activities, and if more than one activity was performed simultaneously a secondary activity was recorded. The surveys also included household and individual questionnaires to gather sociodemographic information about respondents. This study analysed time-use data from all four survey years.

### Ethics approval and consent

The NSO is a state agency responsible for a production of statistical data for national development. The agency is enacted by the Official Information Act, B.E. 2540 (1997) [59]. In accordance with the Official Information Act, participants provided informed consent prior to the survey. and their anonymity and confidentiality of their data were protected by the legislation [59]. For this study, we obtained a permission from the NSO to access microdata of four time-use surveys for research purposes only.

### Participants

This study included only adult participants (18–59 years old) with complete 24-h data. We applied this age range based on the Thai Labour Protection Act (No. 6), B.E. 2560 (2017), which specifies the retirement age for adults at 60 years and above [62]. Retirement is a major event in life that very often significantly affects time-use patterns, including PA, SB, and sleep, as well as many other components of time use [63]. The compulsory retirement age is, therefore, commonly considered as the cut-off point between adulthood and older adulthood. Additionally, Thai 24-h movement recommendations are different for adults and older adults, and the age threshold for older adults is 60 years [13]. The total pooled sample size from four survey years was 167,577 (*n* = 37,702 [50.2% females] in 2001; *n* = 37,544 [49.9% females] in 2004; *n* = 45,751 [50.8% females] in 2009; and *n* = 46,580 [51.0% females] in 2015).

### Measures and data processing

The self-reported time-use data were coded using the ICATUS classification. The time-use codes from the survey year 2001 were based on the Draft ICATUS comprising 10 major groups of activities [57]. The other survey years used the Trial ICATUS consisting of 15 major groups of activities [55, 56, 58]. The Trial ICATUS is a revised version of the Draft ICATUS [61]. One author (NL) harmonised activities in the Draft ICATUS with corresponding activities from the Trial ICATUS. Once all activities were matched, they were categorised into sleep, SB, LPA, and MVPA based on the classification system previously developed for the Trial ICATUS [64]. A detailed description of the method used to classify ICATUS activities can be found elsewhere [64]. Due to insufficient information provided for occupational and travel-related activities in ICATUS, this classification system did not apply to such activities. Instead, the reported occupational and travel-related activities were linked with additional information on occupations and location or mode of travel of respondents available in the household questionnaires, according to the procedures suggested in previous studies [41, 64, 65]. The occupations of respondents were linked with a summary MET previously assigned to the occupation list of 2008 International Standard Classification of Occupations (ISCO-08) [66]. For travel-related codes, the location or mode of travel of respondents was linked with a summary MET assigned to travelling modes available in a previous study [65].

Once all ICATUS-based activities from 2001, 2004, 2009, and 2015 were categorised into sleep, SB, LPA, and MVPA, the time (in minutes) spent in each of the behaviours was calculated. The time-use surveys only captured 1 day of data for each participant, which was assumed to represent their average daily behaviour. Each participant was categorised as either “meeting” or “not meeting” the Thai 24-h movement guidelines for adults. The guidelines include the following recommendations: 1) to engage in at least 150 min/week of moderate PA, or 75 min/week of vigorous PA, or an equivalent combination of the two PA intensities; 2) to interrupt SB every 2 h; and 3) to sleep between 7 and 9 h per day [13]. The guidelines also include an MVPA recommendation for additional health benefits, defined as at least 300 min/week of moderate PA, or 150 min/week of vigorous PA, or an equivalent combination of the two PA intensities [13]. The participants who met all three recommendations for MVPA, SB, and sleep, were categorised as meeting the overall 24-h movement guidelines.

Given that several ICATUS activity groups are broad, it is not feasible to distinguish activities of moderate and vigorous intensity [64]. Following the previously developed classification of ICATUS activities [64], a combination of moderate and vigorous intensities (i.e., MVPA) was used. The amount of time spent in MVPA, SB, and sleep (including napping) was calculated and used to determine whether participants met the recommendations.

For meeting the MVPA recommendation, participants who engaged in a minimum of 150 min/week of MVPA, which translates into an average of approximately 22 min/day, were categorised as meeting the MVPA recommendation. For the SB recommendation, participants who spent no more than 120 min in a single SB activity or consecutive SB activities were classified as meeting the recommendation. From the available data, it was clear that these participants did not engage in SB for more than 120 consecutive minutes, because their SB was interrupted by either PA or sleep. This, however, provides a conservative estimate of the prevalence of meeting the SB guideline, because no data was available on possible interruptions of SB within the 10-min reference periods. Finally, participants who slept between 420 and 540 min per day were classified as meeting the sleep recommendation.

Recommendation for muscle-strengthening activities is also a part of the guidelines [13]. However, in ICATUS-based time-use surveys, these activities are broadly grouped in fitness-related category which includes yoga and other aerobic activities [60]. It was, therefore, not possible to determine whether participants met the recommendation for muscle-strengthening activities.

### Study correlates

Sociodemographic variables were selected a priori based on previous evidence of their associations with PA, SB, and sleep in adults [54, 67–74] and availability of data from the Thai National Time-Use Surveys. We extracted self-reported data on eight sociodemographic variables that were available in all four survey years. These included sex (male, female), age groups (18–29 years, 30–39 years, 40–49 years, and 50–59 years), region (Bangkok, Central, North, North-east, and South), household area (municipal or urban area, non-municipal or rural area), employment status (employed, unemployed), highest education level (none, primary school, secondary school, higher education, and unspecified), marital status (never married, married, and formerly married), and religion (Buddhist, non-Buddhist). Our decision to examine religion as a potential correlate was based on the Kim and Sobal [73] study that found a significant association between religious denomination and PA, and on the mapping review by Langøien et al. [74] that suggested more studies are needed to investigate how different religious affiliations are associated with PA and SB.

### Data analysis

All data were weighted using the population weights provided by NSO, to represent the Thai adult population. Additional weights were used to adjust for slight discrepancies from the assumed uniform distribution of surveys across the 7 days of the week. Percentages (and their 95% confidence intervals [CIs]) of participants meeting MVPA, SB, Sleep, and overall 24-h movement recommendations were computed for the overall sample and by the sociodemographic categories. A series of multivariate logistic regressions was performed to examine the associations between the sociodemographic variables and participants’ compliance within the 24-h movement guidelines. The odds ratios (ORs) were adjusted for sex, age groups, household area, region, marital status, religion, employment status, and education level, and their 95% CIs were calculated. A series of mixed-effects meta-regression analyses with Restricted Maximum Likelihood Estimation (REML) were used to establish the time-trends in prevalence estimates and the time-trends in the associations of sociodemographic variables with meeting the 24-h movement guidelines. Linear and quadratic functions were fitted to each time-trend, and the model with the lower Akaike Information Criterion (AIC) value was selected. The statistical significance level was set at *p* <  0.05. All analyses were conducted in R (R Foundation for Statistical Computing, Vienna, Austria), using “questionr” and “metafor” packages [75, 76].

## Results

### Sample characteristics

The weighted sample included nearly equal males and females (Table 1). In 2001, the most represented age group were young adults (18–29 years old), but the prevalence of older age groups continuously increased throughout the 15-year period. In the first three survey rounds, around two thirds of participants lived in rural areas. From 2009 to 2015 there was a large reallocation from rural to urban areas, resulting in a nearly equal split in the final survey year. In 2001, the most represented region in the sample was North-East (33.8%), while the largest percentage of participants in 2015 were from the Central region (30.4%). In all survey years, most participants were married, Buddhist, and employed. In 2001, primary school was the highest level of education for most participants (60.2%). However, the level of education attainment increased during the study period, as more people completed secondary or higher education.

**Table 1:** Characteristics of the weighted samples in the 2001, 2004, 2009, and 2015 Thai time-use surveys

Sociodemographic variable	2001	2004	2009	2015
**Total** (*n*)	37,702	37,544	45,751	46,580
**Sex** (%)				
Male	49.8	50.1	49.2	49.0
Female	50.2	49.9	50.8	51.0
**Age groups (%)**				
18–29	35.4	34.3	29.6	27.5
30–39	27.4	27.2	26.2	24.5
40–49	22.5	22.8	25.5	26.1
50–59	14.7	15.7	18.7	22.0
**Household area (%)**				
Urban	32.3	34.6	32.7	46.9
Rural	67.7	65.4	67.3	53.1
**Region (%)**				
Bangkok	12.1	14.2	11.1	15.0
Central	22.9	23.7	24.5	30.4
North	19.0	18.2	18.3	16.3
North-East	33.8	31.6	32.8	25.2
South	12.3	12.2	13.2	13.2
**Marital status (%)**				
Never married	24.4	24.6	23.1	27.7
Married	69.7	69.6	70.1	64.5
Formerly married	5.9	5.7	6.8	7.8
**Religion (%)**				
Buddhist	95.1	95.3	95.1	95.4
Non-Buddhist	4.9	4.7	4.9	4.6
**Employment status (%)**				
Employed	82.4	82.4	83.1	80.8
Unemployed	17.6	17.6	16.9	19.2
**Highest education level (%)**				
None	3.2	3.1	4.5	9.8
Primary	60.2	54.6	47.9	35.2
Secondary	25.7	29.2	30.0	31.5
Higher education	10.7	12.7	17.3	23.0
Unspecified	0.2	0.3	0.2	0.5

### Prevalence of meeting 24-h movement guidelines

**Fig. 1:**
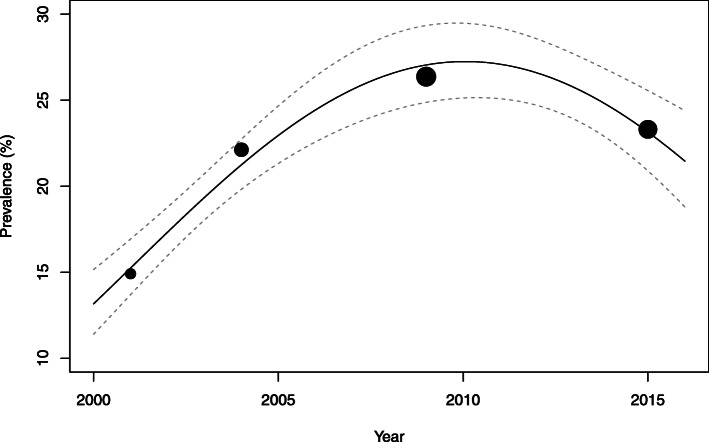
The trend in prevalence of meeting the 24-h movement guidelines among females. The size of the circles is proportional to the precision of each survey year’s estimate of the prevalence

**Fig. 2:**
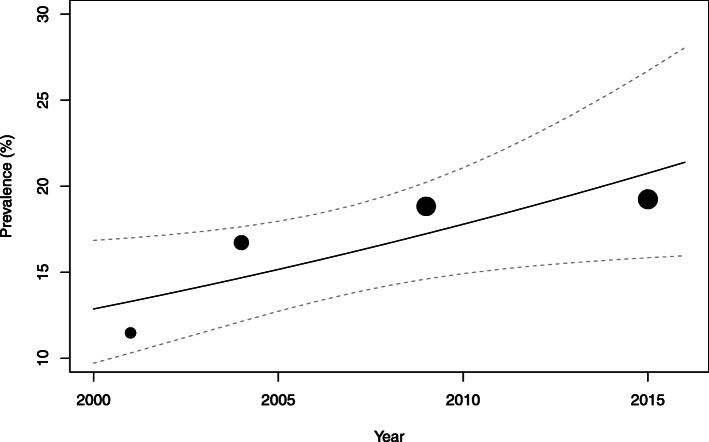
The trend in prevalence of meeting the 24-movement guidelines among males. The size of the circles is proportional to the precision of each survey year’s estimate of the prevalence

In 2015, the prevalence of Thai adults who met 24-h movement guidelines was 21.3% (95% CI: 20.9, 21.7). For the whole sample and for most sociodemographic groups, the sample prevalence rose from 2001 to its peak in 2009, and then declined in 2015. However, this inverted U-shaped trend was found to be significant only among females (Fig. 1), those who were 30–39 years of age, residents of rural areas, inhabitants of the Central and North-East region, those who have never been married or who were formerly married, unemployed, and those who had secondary or higher education. The prevalence of meeting 24-h guidelines showed significant incremental increase from 2001 to 2015 (*p*-value for linear trend < 0.05) among males (Fig. 2), those aged 40–49 years, and those who lived in Bangkok or the South region.

### Associations of sociodemographic characteristics with meeting the overall 24-h movement guidelines

Females had 53, 60, 73, and 43% higher odds of meeting the overall guidelines than males in 2001, 2004, 2009, and 2015, respectively (Table 2). Thai adults who lived in Bangkok had the lowest odds of meeting the overall guidelines, while those from the North region had the highest odds in all survey years except in 2009. In most survey years, significantly higher odds of meeting the overall guidelines were also found for those who were married or formerly married (compared to those who have never been married) and employed (compared to unemployed). An inverted U-shaped trend was found for the odds of meeting the guidelines for the higher education group, where the odds increased from 2001 to 2009 and then decreased in 2015 (*p* = 0.046).

**Table 2:** Meeting the overall guidelines: population prevalence and associations with sociodemographic variables

Sociodemographic variable	Percentage (95% CI)					Adjusted OR (95% CI)				
	2001	2004	2009	2015	*p*-value	2001	2004	2009	2015	*p*-value
**Total (*n*)**	13.2 (12.9–13.5)	19.4 (19.0–19.8)	22.7 (22.3–23.0)	21.3 (20.9–21.7)	0.093*					
**Sex**										
Male	11.5 (11.0–11.9)	16.7 (16.2–17.3)	18.8 (18.3–19.3)	19.2 (18.7–19.8)	0.033*	Ref				
Female	14.9 (14.4–15.4)	22.1 (21.5–22.7)	26.4 (25.8–26.9)	23.3 (22.8–23.9)	< 0.001**	1.53 (1.44–1.63)	1.60 (1.51–1.69)	1.73 (1.65–1.81)	1.43 (1.36–1.50)	0.748*
**Age**										
18–29	11.4 (10.9–12.0)	17.9 (17.2–18.6)	20.0 (19.3–20.6)	17.9 (17.3–18.6)	0.219*	Ref				
30–39	14.7 (14.0–15.4)	20.8 (20.0–21.6)	25.7 (24.9–26.4)	23.7 (22.9–24.5)	< 0.001**	1.00 (0.92–1.09)	0.90 (0.84–0.97)	1.14 (1.07–1.22)	1.11 (1.03–1.18)	0.497*
40–49	14.7 (14.0–15.5)	20.4 (19.5–21.2)	23.1 (22.3–23.9)	23.2 (22.4–23.9)	0.034*	0.94 (0.86–1.03)	0.86 (0.79–0.93)	0.96 (0.90–1.03)	1.04 (0.97–1.12)	0.576*
50–59	12.4 (11.6–13.3)	18.9 (17.9–19.9)	22.1 (21.2–23.0)	20.7 (19.9–21.5)	0.100*	0.82 (0.74–0.91)	0.83 (0.75–0.90)	0.98 (0.91–1.06)	0.95 (0.88–1.03)	0.526*
**Household area**										
Urban	9.8 (9.3–10.3)	17.8 (17.2–18.5)	21.6 (20.9–22.2)	21.4 (20.8–21.9)	0.053*	Ref				
Rural	14.9 (14.4–15.3)	20.2 (19.7–20.7)	23.2 (22.7–23.6)	21.3 (20.7–21.8)	< 0.001**	1.19 (1.10–1.30)	0.95 (0.89–1.02)	0.95 (0.90–1.00)	0.94 (0.89–0.98)	0.471*
**Region**										
Bangkok	8.4 (7.6–9.2)	15.1 (14.1–16.1)	17.0 (16.0–18.1)	19.8 (18.9–20.8)	0.011*	Ref				
Central	11.3 (10.7–12.0)	18.4 (17.6–19.2)	22.7 (21.9–23.5)	21.3 (20.6–21.9)	< 0.001**	1.10 (0.96–1.26)	1.20 (1.08–1.33)	1.52 (1.39–1.68)	1.09 (1.01–1.18)	0.951*
North	16.3 (15.5–17.2)	23.8 (22.8–24.8)	24.4 (23.5–25.3)	22.9 (21.9–23.8)	0.288*	1.50 (1.30–1.73)	1.67 (1.49–1.86)	1.69 (1.52–1.87)	1.25 (1.14–1.36)	0.454*
North-East	15.0 (14.4–15.7)	20.7 (19.9–21.4)	24.5 (23.8–25.1)	21.7 (20.9–22.4)	< 0.001**	1.38 (1.21–1.59)	1.34 (1.21–1.49)	1.75 (1.59–1.93)	1.19 (1.10–1.29)	0.707*
South	11.6 (10.7–12.6)	16.4 (15.4–17.5)	20.2 (19.2–21.2)	20.6 (19.6–21.6)	0.010*	1.02 (0.87–1.20)	1.03 (0.91–1.17)	1.30 (1.16–1.45)	1.05 (0.96–1.16)	0.875*
**Marital status**										
Never married	8.2 (7.7–8.8)	12.9 (12.2–13.6)	17.0 (16.2–17.7)	16.6 (16.0–17.3)	< 0.001**	Ref				
Married	15.0 (14.6–15.5)	21.9 (21.4–22.4)	24.4 (23.9–24.8)	23.3 (22.8–23.7)	0.113*	1.49 (1.36–1.64)	1.65 (1.53–1.79)	1.38 (1.30–1.47)	1.34 (1.26–1.43)	0.509*
Formerly married	12.3 (11.0–13.7)	17.5 (15.9–19.1)	24.1 (22.6–25.6)	21.8 (20.5–23.2)	< 0.001**	1.17 (0.99–1.37)	1.25 (1.09–1.43)	1.31 (1.18–1.46)	1.23 (1.12–1.36)	0.905*
**Religion**										
Buddhist	13.1 (12.8–13.5)	19.6 (19.2–20.0)	22.7 (22.3–23.1)	21.3 (20.9–21.7)	0.103*	Ref				
Non-Buddhist	14.3 (12.7–15.9)	16.5 (14.8–18.2)	21.6 (19.9–23.3)	21.6 (19.8–23.3)	0.003*	1.16 (1.00–1.35)	1.00 (0.86–1.15)	1.12 (0.99–1.25)	1.11 (0.99–1.24)	0.976*
**Employment status**										
Employed	15.0 (14.6–15.4)	21.8 (21.3–22.2)	25.0 (24.6–25.4)	24.0 (23.6–24.4)	0.079*	Ref				
Unemployed	4.8 (4.3–5.3)	8.3 (7.6–9.0)	11.1 (10.4–11.8)	10.0 (9.4–10.6)	< 0.001**	0.28 (0.25–0.32)	0.31 (0.28–0.34)	0.34 (0.31–0.37)	0.34 (0.31–0.36)	0.571*
**Highest education level**										
None	17.1 (15.0–19.2)	17.3 (15.1–19.4)	20.3 (18.6–22.1)	19.8 (18.6–20.9)	0.070*	Ref				
Primary	15.4 (14.9–15.8)	20.7 (20.1–21.2)	22.8 (22.2–23.3)	21.8 (21.2–22.4)	0.112*	0.84 (0.71–0.99)	1.16 (0.99–1.37)	1.01 (0.91–1.14)	1.06 (0.98–1.16)	0.701*
Secondary	9.8 (9.3–10.4)	17.7 (17.0–18.5)	22.2 (21.5–22.9)	21.5 (20.8–22.2)	< 0.001**	0.68 (0.57–0.81)	1.21 (1.03–1.44)	1.15 (1.02–1.29)	1.13 (1.04–1.23)	0.347*
Higher education	7.9 (7.1–8.7)	18.5 (17.4–19.6)	23.7 (22.7–24.6)	20.9 (20.1–21.6)	< 0.001**	0.47 (0.39–0.58)	1.13 (0.95–1.35)	1.26 (1.12–1.43)	1.15 (1.05–1.26)	0.046**
Unspecified	14.8 (7.1–22.5)	14.3 (8.1–20.5)	32.3 (23.7–40.9)	26.5 (20.9–32.0)	0.120*	1.10 (0.55–2.02)	0.92 (0.52–1.54)	1.82 (1.19–2.74)	1.31 (0.97–1.76)	0.639*

*CI* Confidence interval, *OR* Odds ratio adjusted for all other variables in the table, *Ref* Reference group

* = linear model; ** = quadratic model

### Prevalence of meeting the MVPA recommendations

In 2015, the prevalence of Thai adults meeting the MVPA recommendations was 81.7% (95% CI: 81.3, 82.1). For the whole sample, and for all but one sociodemographic group, the sample prevalence of adults meeting the MVPA recommendations was the lowest in 2001, peaked in 2004 or 2009 and then declined in 2015. However, such inverted U-shaped trend was found to be significant only among males, those aged 50–59 years, inhabitants of urban areas, formerly married, non-Buddhists, the unemployed, and those who did not attend school or had secondary education.

The prevalence of Thai adults who met the MVPA recommendations for additional health benefits was 74.3% (95% CI: 73.9, 74. 7) in 2015 (Additional File 1). For the overall sample and for most sociodemographic groups, the sample prevalence was lowest in 2001, rose to its highest point in 2004 and 2009, and then declined in 2015. However, this inverted U-shaped trend was found to be significant only among those who were aged 50–59 years, formerly married, and with unspecified level of education.

### Associations of sociodemographic characteristics with meeting the MVPA recommendations

In all survey years, older age groups (i.e., 40–49 years and 50–59 years) had higher odds of meeting the MVPA recommendations, compared to the youngest adult group (18–29 years; Table 3). Rural residents had 95, 45, 19 and 19% higher odds of meeting the recommendation than those who lived in urban areas in 2001, 2004, 2009, and 2015, respectively (*p*-value for linear trend = 0.042). Those who lived in Bangkok had the lowest odds, while the participants from the North region had the highest odds of meeting the recommendations (in all years except in 2001). An inverted U-shaped trend was found for the odds ratios for the North region, where the odds rose from 2001 to 2009 and then dropped in 2015 (*p* = 0.025). In most survey years, significantly higher odds of meeting the MVPA recommendations were also found for those who were married (compared to those who have never been married), non-Buddhists (compared to Buddhists), and employed (compared to unemployed). Regarding level of education, the lowest odds of meeting the MVPA recommendations were found for those with no formal education.

**Table 3:** Meeting the moderate-to-vigorous physical activity guideline: population prevalence and associations with sociodemographic variables

Sociodemographic variable	Percentage (95% CI)					Adjusted OR (95% CI)				
	2001	2004	2009	2015	*p*-value	2001	2004	2009	2015	*p*-value
**Total (*n*)**	73.2 (72.8–73.7)	88.1 (87.7–88.4)	87.6 (87.3–87.9)	81.7 (81.3–82.1)	0.078**					
**Sex**										
Male	73.7 (73.1–74.4)	89.2 (88.7–89.6)	89.6 (89.2–90.0)	83.6 (83.1–84.0)	0.024**	Ref				
Female	72.7 (72.1–73.3)	87.0 (86.5–87. 5)	85.6 (85.2–86.1)	79.9 (79.4–80.4)	0.151**	1.01 (0.96–1.06)	0.96 (0.90–1.03)	0.74 (0.70–0.78)	0.82 (0.78–0.86)	0.269*
**Age**										
18–29	67.9 (67.1–68.7)	85.0 (84.4–85.6)	83.9 (83.2–84.5)	77.5 (76.8–78.2)	0.127**	Ref				
30–39	73.4 (72.6–74.3)	88.4 (87.8–89.1)	86.7 (86.1–87.3)	80.2 (79.5–81.0)	0.171**	1.18 (1.10–1.26)	1.04 (0.95–1.13)	1.19 (1.10–1.29)	1.06 (0.99–1.13)	0.811*
40–49	77.3 (76.4–78.2)	91.0 (90.4–91.6)	90.6 (90.1–91.2)	84.3 (83.7–85.0)	0.054**	1.36 (1.26–1.47)	1.38 (1.25–1.54)	1.70 (1.55–1.86)	1.24 (1.15–1.33)	0.818*
50–59	79.5 (78.4–80.5)	89.7 (88.9–90.5)	90.4 (89.8–91.0)	85.5 (84.8–86.1)	0.005**	1.48 (1.36–1.62)	1.23 (1.10–1.39)	1.71 (1.55–1.89)	1.31 (1.21–1.42)	0.887*
**Household area**										
Urban	57.6 (56.8–58.5)	81.2 (80.5–81.8)	82.4 (81.8–83.0)	77.3 (76.7–77.8)	0.034**	Ref				
Rural	80.8 (80.3–81.3)	91.6 (91.3–92.0)	90.0 (89.7–90.3)	85.6 (85.2–86.1)	0.237**	1.95 (1.84–2.07)	1.45 (1.33–1.57)	1.19 (1.10–1.28)	1.19 (1.12–1.26)	0.042*
**Region**										
Bangkok	49.9 (48.4–51.3)	73.0 (71.7–74.2)	73.3 (72.1–74.6)	67.7 (66.6–68.8)	0.069**	Ref				
Central	67.5 (66.6–68.5)	86.7 (86.0–87.4)	84.0 (83.3–84.7)	78.6 (77.9–79.3)	0.278**	1.21 (1.11–1.31)	1.80 (1.62–1.99)	1.76 (1.61–1.94)	1.58 (1.47–1.70)	0.497*
North	78.7 (77.8–79.7)	93.1 (92.5–93.7)	92.7 (92.2–93.3)	89.3 (88.6–90.0)	0.107**	1.82 (1.66–1.99)	3.54 (3.11–4.03)	4.12 (3.66–4.64)	3.46 (3.14–3.81)	0.025**
North-East	82.0 (81.3–82.7)	91.7 (91.2–92.2)	90.7 (90.3–91.2)	85.9 (85.3–86.5)	0.122**	2.21 (2.02–2.41)	2.73 (2.44–3.06)	3.20 (2.88–3.55)	2.48 (2.29–2.70)	0.708*
South	74.9 (73.6–76.1)	90.9 (90.0–91.7)	90.3 (89.6–91.0)	87.3 (86.5–88.2)	0.140**	1.49 (1.35–1.65)	2.49 (2.17–2.87)	2.92 (2.59–3.30)	2.77 (2.51–3.06)	0.080*
**Marital status**										
Never married	65.4 (64.5–66.4)	82.6 (81.8–83.4)	82.7 (82.0–83.4)	76.9 (76.1–77.6)	0.056**	Ref				
Married	76.0 (75.4–76.5)	90.4 (89.7–90.4)	89.2 (88.8–89.5)	83.7 (83.3–84.1)	0.119**	0.97 (0.91–1.04)	1.24 (1.14–1.34)	1.22 (1.13–1.31)	1.13 (1.06–1.20)	0.664*
Formerly married	73.1 (71.3–75.0)	86.9 (85.8–88.3)	87.5 (86.3–88.6)	82.5 (81.2–83.7)	0.024**	0.82 (0.73–0.92)	0.95 (0.81–1.11)	1.09 (0.96–1.24)	1.04 (0.93–1.15)	0.477*
**Religion**										
Buddhist	72.9 (72.5–73.4)	88.0 (87.6–88.3)	87.4 (87.1–87.7)	81.5 (81.1–81.8)	0.082**	Ref				
Non-Buddhist	78.8 (77.0–80.7)	90.4 (89.0–91.7)	90.7 (89.5–91.9)	86.3 (84.9–87. 8)	0.020**	1.44 (1.27–1.63)	1.2 (1.01–1.45)	1.49 (1.28–1.75)	1.25 (1.09–1.43)	0.831*
**Employment status**										
Employed	74.8 (74.3–75.3)	90.5 (90.2–90.8)	89.1 (88.8–89.4)	83.3 (82.9–83.7)	0.168**	Ref				
Unemployed	65.6 (64.5–66.8)	76.6 (75.6–77.6)	80.1 (79.3–81.0)	75.0 (74.1–75.9)	< 0.001**	0.74 (0.69–0.78)	0.41 (0.38–0.44)	0.59 (0.55–0.63)	0.63 (0.59–0.66)	0.947*
**Highest education level**										
None	79.9 (77.6–82.2)	84.4 (82.4–86.5)	84.6 (83.1–86.2)	78.6 (77.4–79.8)	< 0.001**	Ref				
Primary	80.7 (80.2–81.2)	91.0 (90.6–91.4)	89.9 (89.5–90.3)	86.6 (86.1–87.1)	0.707*	1.06 (0.91–1.22)	1.67 (1.40–1.99)	1.39 (1.22–1.59)	1.54 (1.41–1.68)	0.460*
Secondary	63.2 (62.2–64.1)	84.5 (83.8–85.2)	86.4 (85.8–86.9)	80.3 (79.6–80.9)	0.009**	0.62 (0.53–0.72)	1.45 (1.21–1.74)	1.42 (1.24–1.62)	1.21 (1.12–1.32)	0.113**
Higher education	53.7 (52.1–55.2)	84.5 (83.5–85.6)	83.7 (82.9–84.5)	77.6 (76.8–78.3)	0.125**	0.44 (0.38–0.52)	1.43 (1.18–1.72)	1.38 (1.20–1.59)	1.27 (1.16–1.39)	0.122**
Unspecified	56.1 (45.3–66.8)	89.1 (83.6–94.6)	87.5 (81.5–93.6)	76.9 (71.6–82.2)	0.085**	0.51 (0.32–0.82)	2.87 (1.62–5.49)	1.46 (0.84–2.71)	1.04 (0.76–1.42)	0.558**

*CI* Confidence interval, *OR* Odds ratio adjusted for all other variables in the table, *Ref* Reference group

* = linear model; ** = quadratic model

In all survey years, males had higher odds of meeting the MVPA recommendations for additional health benefits, compared to females (Additional File 1). Older ages (30–59 years) also had higher odds of meeting the recommendations than the youngest adult group (18–29 years). The odds of meeting the recommendations were 2.09, 1.54, 1.35, and 1.27 times higher among rural residents, compared with those who lived in urban areas in 2001, 2004, 2009, and 2015, respectively (*p*-value for linear trend = 0.039). Thai adults who lived in Bangkok had the lowest odds of meeting the recommendations, while those who lived in the North region had the highest odds in all survey years except in 2001. In all survey years, higher odds of meeting the recommendations were also found to be significant for those who were non-Buddhists (compared to Buddhists), and employed (compared to unemployed). For the education level, Thai adults who did not attend school had the lowest odds of meeting the recommendations in all survey years except in 2001. An inverted U-shaped trend was found for the odds ratios for those who had higher education, where the odds increased from 2001 to 2009 and then declined in 2015 (*p* = 0.004).

### Prevalence of meeting the SB recommendation

In 2015, the prevalence of Thai adults who met the SB recommendation was 44.6% (95% CI: 44.1, 45.0). A significant linear increase was found for the whole sample, males, all age groups above 30 years of age, those who lived in urban or rural areas, residents of all regions except the North, those who were married and those who have never been married, Buddhists, employed, and those with primary and secondary education. The sample prevalence of adults aged 18–29 years, unemployed, and those with higher education was found to be lowest in 2001, rose to its highest in 2009, and then declined in 2015 (*p*-value for inverted U-shaped trend < 0.05).

### Associations of sociodemographic characteristics with meeting the SB recommendation

In 2001, 2004, 2009, and 2015, females had 23, 47, 85, and 44%, respectively, higher odds of meeting the SB recommendation than males (Table 4). The youngest adult group (18–29 years) had the highest odds of meeting the recommendation compared to the remaining age groups. Those who lived in urban areas had higher odds of meeting the recommendation than those who lived in rural areas in all survey years except in 2001. Significantly higher odds of meeting the SB recommendation were also found among those who were married or formerly married (compared to those who have never been married), and employed (compared to unemployed). For those who had higher education, the odds of meeting the SB recommendation rose from 2001 to 2009 and dropped in 2015 (quadratic trend *p* = 0.048).

**Table 4:** Meeting the sedentary behaviour guideline: population prevalence and associations with sociodemographic variables

Sociodemographic variable	Percentage (95% CI)					Adjusted OR (95% CI)				
	2001	2004	2009	2015	*p*-value	2001	2004	2009	2015	*p*-value
**Total (*n*)**	36.9 (36.5–37.4)	39.6 (39.1–40.1)	44.7 (44.2–45.1)	44.6 (44.1–45.0)	0.002*					
**Sex**										
Male	35.7 (35.0–36.4)	36.4 (35.7–37.0)	38.5 (37.8–39.1)	41.7 (41.0–42.3)	< 0.001*	Ref				
Female	38.2 (37.5–38.9)	42.9 (42.2–43.6)	50.7 (50.1–51.4)	47.4 (46.8–48.0)	0.079*	1.23 (1.18–1.29)	1.47 (1.40–1.53)	1.85 (1.78–1.93)	1.44 (1.38–1.50)	0.147**
**Age**										
18–29	34.1 (33.3–34.9)	37.9 (37.1–38.7)	41.8 (41.0–42.6)	41.8 (40.9–42.6)	< 0.001**	Ref				
30–39	39.2 (38.3–40.1)	42.6 (41.7–43.6)	49.8 (48.9–50.7)	48.0 (47.1–48.9)	0.025*	0.88 (0.83–0.94)	0.88 (0.83–0.94)	1.07 (1.01–1.13)	0.95 (0.90–1.00)	0.632*
40–49	39.3 (38.3–40.4)	40.2 (39.2–41.3)	44.1 (43.2–45.0)	45.6 (44.7–46.5)	< 0.001*	0.83 (0.78–0.89)	0.77 (0.72–0.82)	0.80 (0.75–0.85)	0.80 (0.75–0.85)	0.952*
50–59	35.9 (34.6–37.2)	37.3 (36.1–38.5)	42.9 (41.9–44.0)	43.1 (42.1–44.1)	0.001*	0.76 (0.71–0.82)	0.70 (0.65–0.75)	0.81 (0.75–0.86)	0.76 (0.71–0.81)	0.856*
**Household area**										
Urban	34.9 (34.1–35.7)	38.3 (37.4–39.1)	45.5 (44.7–46.3)	45.2 (44.6–45.9)	0.002*	Ref				
Rural	37.9 (37.3–38.5)	40.3 (39.7–40.9)	44.3 (43.7–44.8)	44.0 (43.4–44.6)	0.005*	0.96 (0.91–1.02)	0.93 (0.87–0.98)	0.90 (0.86–0.95)	0.94 (0.90–0.98)	0.935*
**Region**										
Bangkok	35.1 (33.7–36.5)	35.6 (34.3–36.9)	44.2 (42.8–45.6)	46.4 (45.2–47.6)	< 0.001*	Ref				
Central	35.1 (34.1–36.1)	39.5 (38.5–40.5)	45.9 (44.9–46.8)	44.2 (43.4–45.0)	0.029*	0.89 (0.82–0.97)	1.11 (1.02–1.20)	1.12 (1.04–1.21)	0.85 (0.80–0.91)	0.677*
North	39.4 (38.3–40.6)	46.3 (45.1–47.4)	46.5 (45.5–47.6)	46.8 (45.7–47.9)	0.137*	0.98 (0.90–1.08)	1.46 (1.34–1.59)	1.16 (1.06–1.25)	0.99 (0.92–1.07)	0.623*
North-East	38.2 (37.4–39.1)	39.0 (38.2–39.9)	43.2 (42.5–44.0)	42.4 (41.5–43.3)	0.022*	0.96 (0.88–1.05)	1.04 (0.96–1.13)	1.03 (0.95–1.12)	0.84 (0.78–0.90)	0.548*
South	35.0 (33.6–36.4)	35.9 (34.5–37.3)	44.0 (42.7–45.2)	44.8 (43.6–46.1)	0.001*	0.85 (0.77–0.94)	0.95 (0.86–1.05)	1.03 (0.94–1.12)	0.90 (0.83–0.98)	0.878*
**Marital status**										
Never married	26.9 (26.0–27.8)	30.8 (29.9–31.7)	36.5 (35.5–37.4)	37.1 (36.3–37.9)	0.001*	Ref				
Married	40.3 (39.8–40.9)	42.6 (42.0–43.2)	46.7 (46.2–47.3)	47.4 (46.8–48.0)	< 0.001*	1.62 (1.52–1.72)	1.52 (1.43–1.61)	1.41 (1.34–1.48)	1.44 (1.37–1.51)	0.609*
Formerly married	38.6 (36.6–40.7)	40.8 (38.7–42.9)	51.3 (49.6–53.1)	47.8 (46.1–49.4)	0.070*	1.52 (1.36–1.69)	1.45 (1.30–1.61)	1.63 (1.49–1.78)	1.48 (1.36–1.60)	0.993*
**Religion**										
Buddhist	37.0 (36.5–37.5)	39.9 (39.3–40.4)	44.6 (44.2–45.1)	44.6 (44.2–45.1)	0.002*	Ref				
Non-Buddhist	36.7 (34.5–38.9)	35.1 (32.9–37.3)	45.8 (43.7–47.8)	43.9 (41.8–46.0)	0.066*	0.99 (0.89–1.10)	0.92 (0.82–1.03)	1.07 (0.97–1.17)	0.95 (0.87–1.05)	0.980*
**Employment status**										
Employed	40.7 (40.2–41.3)	43.2 (42.6–43.7)	48.0 (47.5–48.5)	48.8 (48.3–49.3)	< 0.001*	Ref				
Unemployed	18.9 (18.0–20.0)	23.0 (22.0–24.1)	28.2 (27.2–29.2)	26.9 (26.0–27.8)	< 0.001**	0.34 (0.31–0.36)	0.37 (0.35–0.39)	0.36 (0.34–0.39)	0.36 (0.34–0.38)	0.819*
**Highest education level**										
None	45.1 (42.3–47.9)	41.3 (38.5–44.1)	43.5 (41.3–45.6)	43.3 (41.9–44.8)	0.817*	Ref				
Primary	40.1 (39.5–40.8)	41.8 (41.2–42.5)	44.9 (44.3–45.6)	45.6 (44.8–46.3)	< 0.001*	0.70 (0.62–0.79)	0.94 (0.83–1.07)	0.96 (0.87–1.05)	1.08 (1.01–1.15)	0.215*
Secondary	31.9 (31.0–32.8)	37.1 (36.2–38.0)	44.7 (43.8–45.5)	46.8 (46.0–47.6)	< 0.001*	0.59 (0.52–0.67)	0.88 (0.78–1.01)	1.01 (0.92–1.11)	1.13 (1.06–1.21)	0.060*
Higher education	28.8 (27.4–30.2)	35.6 (34.2–36.9)	44.0 (42.9–45.1)	40.2 (39.3–41.1)	< 0.001**	0.43 (0.38–0.50)	0.73 (0.63–0.84)	0.96 (0.86–1.06)	0.90 (0.84–0.97)	0.048**
Unspecified	33.1 (22.9–43.3)	34.9 (26.5–43.3)	73.1 (65.0–81.3)	62.3 (56.2–68.4)	0.083*	0.58 (0.35–0.93)	0.75 (0.50–1.11)	3.13 (2.04–4.90)	1.83 (1.40–2.41)	0.061**

*CI* Confidence interval, *OR* Odds ratio adjusted for all other variables in the table, *Ref* Reference group

* = linear model; ** = quadratic model

### Prevalence of meeting the sleep recommendation

In 2015, the prevalence of Thai adults who met the sleep recommendation was 56.4% (95% CI: 56.0, 56.9). For the overall sample and for most sociodemographic groups, the sample prevalence of adults meeting the sleep recommendation was lowest in 2001, rose to its highest in 2009, and then declined in 2015. However, an inverted U-shaped trend was found to be significant only in the overall sample, among Buddhists, and among those who had secondary or higher education.

### Associations of sociodemographic characteristics with meeting the sleep recommendation

Females had 28, 25, 32, and 35% higher odds of meeting the sleep recommendation than males in 2001, 2004, 2009, and 2015, respectively (Table 5). In most survey years, significantly higher odds of meeting the recommendation were found for the youngest adult group (compared to those aged 50–59 years), inhabitants of the North-East region (compared to those from Bangkok), married (compared to those who have never been married), and employed (compared to unemployed). Thai adults without formal education had the lowest odds of meeting the sleep recommendation.

**Table 5:** Meeting the sleep guideline: population prevalence and associations with sociodemographic variables

Sociodemographic variable	Percentage (95% CI)					Adjusted OR (95% CI)				
	2001	2004	2009	2015	*p*-value	2001	2004	2009	2015	*p*-value
**Total (*n*)**	53.7 (53.2–54.2)	56.1 (55.6–56.6)	57.8 (57.4–58.3)	56.4 (56.0–56.9)	< 0.001**					
**Sex**										
Male	51.7 (51.0–52.4)	54.5 (53.7–55.2)	55.6 (55.0–56.3)	53.7 (53.0–54.3)	0.494*	Ref				
Female	55.7 (55.0–56.4)	57.8 (57.1–58.5)	60.0 (59.4–60.6)	59.0 (58.4–59.6)	0.085*	1.28 (1.22–1.33)	1.25 (1.20–1.30)	1.32 (1.27–1.37)	1.35 (1.30–1.40)	0.715*
**Age**										
18–29	53.8 (52.9–54.6)	56.2 (55.4–57.1)	58.0 (57.2–58.8)	54.6 (53.8–55.5)	0.798*	Ref				
30–39	56.4 (55.5–57.4)	56.9 (55.9–57.9)	59.6 (58.8–60.5)	58.3 (57.4–59.2)	0.181*	1.10 (1.03–1.16)	0.94 (0.88–0.99)	0.99 (0.94–1.05)	1.02 (0.96–1.07)	0.900*
40–49	53.9 (52.9–55.0)	56.6 (55.6–57.7)	57.6 (56.7–58.5)	58.4 (57.5–59.3)	0.002*	0.99 (0.94–1.06)	0.95 (0.89–1.01)	0.94 (0.89–0.99)	1.07 (1.01–1.13)	0.744*
50–59	48.0 (46.7–49.3)	53.9 (52.6–55.1)	55.5 (54.4–56.5)	54.2 (53.2–55.2)	0.182*	0.83 (0.77–0.89)	0.9 (0.84–0.97)	0.93 (0.88–0.99)	0.96 (0.91–1.03)	0.563*
**Household area**										
Urban	52.1 (51.2–53.0)	57.0 (56.1–57.8)	58.0 (57.2–58.8)	58.2 (57.5–58.9)	0.065*	Ref				
Rural	54.5 (53.9–55.1)	55.7 (55.1–56.3)	57.8 (57.2–58.3)	54.8 (54.2–55.4)	0.816*	1.01 (0.96–1.07)	0.97 (0.92–1.02)	0.97 (0.93–1.02)	0.91 (0.87–0.95)	0.647*
**Region**										
Bangkok	48.3 (46.9–49.8)	57.5 (56.1–58.8)	55.3 (53.9–56.7)	58.7 (57.5–59.8)	0.123*	Ref				
Central	54.9 (53.9–56.0)	55.6 (54.5–56.6)	59.4 (58.5–60.3)	57.4 (56.6–58.2)	0.212*	1.33 (1.23–1.44)	0.94 (0.87–1.01)	1.27 (1.17–1.37)	1.03 (0.96–1.09)	0.633*
North	57.0 (55.9–58.2)	56.0 (54.8–57.1)	56.8 (55.7–57.9)	54.8 (53.7–55.9)	0.103*	1.46 (1.34–1.59)	0.98 (0.90–1.07)	1.16 (1.07–1.26)	0.97 (0.90–1.04)	0.285*
North-East	55.5 (54.6–56.3)	58.0 (57.1–58.9)	60.3 (59.5–61.1)	57.7 (56.8–58.6)	0.434*	1.36 (1.25–1.47)	1.05 (0.97–1.14)	1.38 (1.27–1.49)	1.12 (1.04–1.20)	0.741*
South	46.7 (45.2–48.1)	51.0 (49.5–52.4)	52.5 (51.2–53.7)	51.1 (49.8–52.3)	0.220*	0.93 (0.85–1.02)	0.78 (0.71–0.86)	0.95 (0.87–1.04)	0.81 (0.75–0.87)	0.800*
**Marital status**										
Never married	53.2 (52.2–54.2)	53.0 (52.0–54.0)	56.2 (55.2–57.1)	54.7 (53.9–55.6)	0.229*	Ref				
Married	54.2 (53.6–54.8)	57.6 (57.0–58.2)	58.8 (58.2–59.3)	57.5 (57.0–58.1)	0.250*	0.98 (0.93–1.04)	1.20 (1.13–1.27)	1.10 (1.04–1.16)	1.10 (1.05–1.15)	0.814*
Formerly married	49.9 (47.8–52.0)	52.1 (50.0–54.2)	54.0 (52.3–55.8)	53.2 (51.6–54.8)	0.101*	0.85 (0.77–0.94)	1.00 (0.90–1.11)	0.91 (0.84–0.99)	0.93 (0.85–1.00)	0.924*
**Religion**										
Buddhist	53.9 (53.3–54.4)	56.4 (55.9–56.9)	58.0 (57.6–58.5)	56.6 (56.1–57.0)	< 0.001**	Ref				
Non-Buddhist	50.7 (48.4–53.0)	51.2 (48.9–53.5)	54.2 (52.1–56.2)	53.3 (51.2–55.4)	0.070*	1.07 (0.97–1.19)	1.00 (0.90–1.11)	1.04 (0.95–1.14)	1.08 (0.98–1.18)	0.931*
**Employment status**										
Employed	55.1 (54.6–55.7)	58.2 (57.6–58.7)	60.1 (59.6–60.6)	58.8 (58.4–59.3)	0.149*	Ref				
Unemployed	46.9 (45.7–48.2)	46.6 (45.4–47.8)	47.0 (45.9–48.1)	46.1 (45.1–47.2)	0.353*	0.69 (0.65–0.73)	0.60 (0.56–0.63)	0.54 (0.51–0.57)	0.55 (0.53–0.58)	0.351*
**Highest education level**										
None	45.0 (42.2–47.8)	44.7 (41.9–47.5)	50.2 (48.0–52.3)	52.3 (50.9–53.8)	< 0.001*	Ref				
Primary	53.9 (53.3–54.5)	55.3 (54.6–56.0)	55.8 (55.1–56.4)	54.6 (53.8–55.3)	0.695*	1.41 (1.25–1.59)	1.40 (1.24–1.58)	1.14 (1.04–1.25)	1.05 (0.98–1.13)	0.261*
Secondary	54.0 (53.0–55.0)	57.1 (56.2–58.1)	59.4 (58.5–60.2)	56.6 (55.8–57.4)	< 0.001**	1.53 (1.35–1.73)	1.68 (1.48–1.90)	1.42 (1.29–1.56)	1.19 (1.11–1.27)	0.275*
Higher education	54.5 (52.9–56.0)	60.1 (58.7–61.5)	63.2 (62.1–64.2)	60.8 (59.9–61.8)	< 0.001**	1.48 (1.29–1.69)	1.73 (1.51–1.98)	1.67 (1.51–1.85)	1.43 (1.33–1.54)	0.740*
Unspecified	58.2 (47.5–68.9)	63.1 (54.5–71.6)	53.0 (43.8–62.2)	50.9 (44.6–57.2)	0.045*	1.92 (1.22–3.06)	2.02 (1.38–3.00)	1.07 (0.73–1.58)	0.86 (0.66–1.11)	0.091*

*CI* Confidence interval, *OR* Odds ratio adjusted for all other variables in the table, *Ref* Reference group

* = linear model; ** = quadratic model

## Discussion

The present study was the first to determine 15-year trends and sociodemographic correlates of meeting the new integrated 24-h movement guidelines using nationally representative time-use data. From these self-reported data, we found that only one in five Thai adults met the overall 24-h movement guidelines. However, the majority of Thai adults met the MVPA recommendation, slightly above half met the sleep recommendation, and slightly below half met the SB recommendation. Moreover, in contrast to our hypotheses, there was an increase over time in the number of Thai adults who met the SB recommendation, while the prevalence of meeting the MVPA and sleep recommendations peaked in 2004 and 2009 and slightly declined in 2015.

Our findings suggest that changes in movement behaviours over time among Thai adults do not align with the global trends [39, 52–54, 77]. We analysed the trends in SB based on the percentage of adults who do not engage in long periods of uninterrupted SB, and, therefore, our findings are not directly comparable with SB trends from previous studies where the overall duration of SB was analysed. The rise in the prevalence of meeting the MVPA (from 2001 to 2004) and SB recommendations may be due to the establishment of national organizations to promote PA in Thailand in the early 2000s [47]. The reasons for a decline in MVPA since 2009 might be associated with Thailand’s economic development and its transition to an upper-middle income country in 2011. This has coincided with a transition towards a more urban society [78], changes in job characteristics and the living environment, which may result in reduced opportunities to engage in MVPA in everyday life. Insufficient PA is associated with a lower likelihood of meeting the sleep recommendations [79–82], which may explain the slight decrease in the prevalence of Thai adults meeting the sleep recommendation in the same period.

In regard to the sociodemographic correlates of meeting the 24-h movement guidelines, employed adults were more likely to meet every recommendation (i.e., MVPA, MVPA for additional health benefits, SB, sleep, and overall 24-h movement guidelines) compared to adults who were unemployed. Thai adults who were married were found to be more likely to meet the MVPA, SB, sleep, and overall 24-h movement guidelines compared to those who have never been married. Thai females tended to meet the SB, sleep, and the overall guidelines more than males. However, we found that the percentage of males meeting the SB and overall guidelines increased over the 15 years. Education level seemed to play an important role in meeting the guidelines: those with no formal education were found to be the least likely to meet the MVPA, sleep, and MVPA recommendations for additional health benefits compared to other education groups. During the study period, the likelihood of meeting the overall 24-h movement guidelines was found to increase significantly for adults who had higher education.

We found that around one in five Thai adults met the overall 24-h movement guidelines (i.e., MVPA, SB, and sleep recommendations combined) in 2015. A recent study found that in the same year only 0.4% of Korean adults met a similar ‘ideal’ combination of movement behaviours [42]. However, a direct comparison between these findings is not possible, as there are differences in methodology used to assess movement behaviours and categorise participants. For example, the Korean study used separate questionnaires to estimate daily MVPA and SB, and a single question for sleep duration (h), rather than a 24-h time-use dairy. The overall 24-h movement behaviour guideline in the Korean study included meeting MVPA (defined as ≥600 METs minute/week) and sleep (7–9 h/day) recommendations and not having high sitting time (≥9 h/day). Besides, the Korean study had a higher upper age limit of 64 years compared to 59 years in the current study.

The prevalence of Thai adults meeting the MVPA recommendation was very high in all survey years, even with a decrease in 2009 and 2015. National data collected using PA questionnaires, such as Global Physical Activity Questionnaire (GPAQ) and International Physical Activity Questionnaire (IPAQ), suggest similarly high prevalence rates; 77.5% in 2003, 74.9% in 2009, and 80.8% in 2015 [83]. Our results for MVPA prevalence in the Thai population are concordant with previous findings mainly from low- and middle-income countries For example, the percentage of meeting MVPA recommendation in East and Southeast Asia increased from 74.3% in 2001 to 82.7% in 2016 [77]. In some countries, such as Nepal [84], the prevalence of meeting MVPA recommendation was even higher than in Thailand. In studies that relied on data from PA questionnaires, such high prevalence rates may be a consequence of self-report bias. However, given that the questions in the Thai time-use survey did not ask specifically about PA levels, it is less likely that such bias have affected the results of our study.

The increasing MVPA in the Thai population found in the current study may be a positive result of national efforts to increase population PA. Important events for the promotion of PA in Thailand were the establishment of the Thai Health Promotion Foundation in 2001 and the Division of Physical Activity and Health, Ministry of Public Health in 2002 [47]. These two organizations along with their network of alliances aim to improve the population PA by developing policies and implementing interventions. They have supported nationwide campaigns to promote PA and the development of the first national strategic plan on the promotion of PA (2018–2030) [48].

The process of urbanisation may have had a significant impact on PA level of Thai adults. We found that urban residence in Thailand is associated with lower prevalence of MVPA. Evidence of such association was also found in other adult populations [42, 67, 68, 70]. With increasingly evident changes in the population structure in Thailand, particularly migration of working adults to urban areas, continuing coordinated efforts are needed to prevent a potential decline in population PA. National policies should increase opportunities that support PA specifically in urban settings, such as increasing walkability areas, improving public transportation systems, and enabling safe public use of open green spaces. It is, therefore, essential to incorporate collaborations with non-health sectors such as urban planning and transport infrastructure to create such environments. With changing nature of work, PA undertaken in occupational domain is also reduced. Future efforts are, therefore, needed to promote other types of PA particularly in transport and leisure-time domains.

Our results also indicate significant differences in the likelihood of meeting the MVPA recommendation between sexes, age groups, household areas, employment status, and education levels. Consistent with previous studies, male sex, rural setting, employment, and higher education level were significant correlates of more MVPA [42, 67, 68, 70]. However, in contrast to previous findings, older age groups in Thailand had higher MVPA than the youngest group. This finding adds to the mixed evidence of associations between younger age and higher PA [68, 85], and suggests that more PA promotion interventions might be needed among younger adults. This may be a result of advances in screen entertainment that supports SB and is widely used particularly among younger generations [86]. Future intervention studies may consider exploring possible uses of emerging media and new technologies, which are increasing in popularity among young adults, to promote PA [87].

We found that more than a half of Thai adults do not interrupt their sedentary activities at least every 2 h. It is encouraging that the number of Thai adults who meet the SB recommendation is increasing. This may be due to a rapid development of the body of evidence on detrimental effects of SB that occurred in the first half of the previous decade. The odds of meeting the SB recommendation were significantly higher among Thai females when compared with their male counterparts. This result supports the finding of a recent systematic review on SB correlates in Thailand that male adults engage more in SB than females [85]. This result is, however, contradictory to the finding of a previous review that included studies from other countries [88]. The reason for this difference may be that most previous evidence on this association came from high-income countries. Moreover, in accordance with existing literature, our study confirms that older age, being single, and being unemployed have positive associations with SB [88].

To our knowledge, this study is the first to examine the trends in meeting the sleep recommendation in Thai adults. Only about a half of Thai adults met the sleep recommendation. The prevalence of Thai adults meeting the sleep recommendation increased over the study period (except for a slight decrease in 2015). In comparison, no trends were observed in several other countries [52–54, 89], while a declining trend was found in the American adult population from 2004 to 2012 [90]. In 2015, the prevalence of meeting sleep recommendation among Thai adults was similar to that for Korean adults [42]. This study explored sleep duration only, while sleep quality has not been examined. As there is limited information about trends in sleep among Thai adults, more studies are needed in this area, particularly including measures of different aspects of sleep quality.

### Strengths and limitations

There are several strengths of the present study. First, this is the first study to examine the prevalence of meeting the Thai 24-h movement recommendations. Second, a large sample size ensured adequate statistical power and sufficient precision in estimating population effect size. Third, this study showcases how to employ ICATUS-based data in PA and SB research. Last, we used time-use survey data which have been shown to provide reliable and valid estimates in large-scale studies [23].

This study had limitations. Firstly, ICATUS does not include a detailed breakdown of occupation- and travel-related activities according to their intensity level and posture. A more detailed assessment of these activities would allow for a more precise quantification of PA and SB levels. Using device-based measures of PA and SB might improve the accuracy of prevalence estimates. However, due to time and cost limitations, device-based measurements may be challenging to use for the assessment of PA and SB in large-scale studies, particularly in low- and middle-income countries [36, 91]. Also, the activities were reported in 10-min reference periods for the last three observations. This may have led to an underestimation of the prevalence of meeting the SB guideline because no data was available on possible short interruptions of SB within these reference periods. Secondly, the Thai national time-use surveys were based on a one-day diary. Given that movement behaviours may vary across days of measurement, a longer measurement period would likely provide more reliable individual estimates. Thirdly, the time-use surveys used to determine population-level estimates in this study were collected prior to the release of the Thai 24-h guidelines. The movement behaviours of the Thai population may have changed now that the guidelines have been published. This should be examined in future surveys. Finally, in this study we did not analyse domain-specific data on PA and SB (e.g. at work, in household, in transport, and in leisure-time). Such an additional analysis would provide even deeper understanding of patterns of these behaviours in the Thai population, but it was beyond the scope of this paper.

### Recommendations for future research

Prevalence and correlates of meeting the 24-h guidelines should be comprehensively explored in different countries and world regions, because they may vary across different social, environmental, cultural, and political contexts. Besides the assessment of time use, most time-use surveys include only a limited number of questions on sociodemographic characteristics of participants. Future studies in this area would benefit from investigating a wider range of correlates, beyond only individual-level sociodemographic factors. Future research should also consider: 1) using more detailed questions on the time spent in occupation- and travel-related activities; 2) assessing time use over multiple days; and 3) analysing correlates of domain-specific PA and SB.

## Conclusions

Based on self-reported time-use data, we found that, despite promising trends in the prevalence of meeting PA, SB, and sleep recommendations, a large majority of Thai adults do not meet the overall 24-h movement guidelines. Further actions are needed to promote more MVPA, less SB, and adequate sleep in Thai adults, particularly among males, those living in urban areas, inhabitants of Bangkok and South Thailand, unemployed, and those with low education level. To be able to assess the effectiveness of such actions at the national level, it is critical to maintain regular nationwide surveillance and monitoring systems. Future studies should explore in more detail sleep quality and patterns of PA and SB in the Thai population. Moreover, since the 24-h guidelines were only recently issued in Thailand, it is important to disseminate them, to ensure they reach as many people as possible. Our findings indicate that, nation-wide public health actions are needed to promote more PA, less SB, and adequate sleep among Thai adults, to increase the prevalence of meeting 24-h guidelines.

## Supplementary information

**Additional file 1.** Meeting the MVPA recommendation for additional health benefits. The prevalence and association table of MVPA for additional health benefits.

## Data Availability

The datasets analysed in the current study are available from the NSO, but restrictions apply to the availability of these data. Given that a permission from NSO is needed to access and use the data, they cannot be made publicly available alongside this paper. The data are, however, available from the first author upon a reasonable request and with written permission from the NSO.

## Abbreviations

AIC: Akaike Information Criterion
ATUS: American Time Use Survey
BMI: Body mass index
GPAQ: Global Physical Activity Questionnaire
ICATUS: International Classification of Activities for Time-Use Statistics
IPAQ: International Physical Activity Questionnaire
ISCO: International Standard Classification of Occupations
LPA: Light physical activity
METs: Metabolic equivalents
MVPA: Moderate-to-vigorous physical activity
NSO: National Statistical Office
PA: Physical activity
REML: Restricted Maximum Likelihood Estimation
SB: Sedentary behaviour
GSS-TU: Statistics Canada’s General Social Survey – Time Use
WHO: World Health Organization
