# Supplementary material for: Trends and correlates of meeting 24-hour movement guidelines: a 15-year study among 167,577 Thai adults
Source: Int J Behav Nutr Phys Act. 2020 Aug 24;17:106. doi: 10.1186/s12966-020-01011-9 (PMC7446156; doi:10.1186/s12966-020-01011-9)
Supplement: Supplementary file 1 — Additional file 1. Meeting the MVPA recommendation for additional health benefits. The prevalence and association table of MVPA for additional health benefits. [file 12966_2020_1011_MOESM1_ESM.pdf]

Meeting the moderate-to-vigorous physical activity guideline for additional health benefits: population prevalence and associations with sociodemographic variables

Meeting Enhance MVPA Guidelines	Prevalence (95% CI)					Adjusted OR (95% CI)				
	2001	2004	2009	2015	P-value	2001	2004	2009	2015	P-value
**Total (*n*)**	66.7 (66.3 - 67.2)	82.7 (82.3 - 83.0)	81.0 (80.6 - 81.3)	74.3 (73.9 - 74.7)	0.152**					
**Sex (%)**										
Male	69.3 (68.7 – 70.0)	85.3 (84.8 - 85.8)	84.4 (83.9 - 84.9)	77.6 (77.1 - 78.15)	0.095**					
Female	64.1 (63.5 - 64.8)	80.0 (79.4 - 80.5)	77.7 (77.1 - 78.2)	71.1 (70.5 - 71.7)	0.827*	0.85 (0.81 - 0.89)	0.8 (0.76 - 0.85)	0.7 (0.67 - 0.74)	0.75 (0.72 - 0.79)	0.5308*
**Age (%)**										
18 - 29	60.6 (59.8 - 61.4)	78.4 (77.7 - 79.1)	75.5 (74.8 - 76.2)	69.98 (68.2 - 69.8)	0.791*	Ref				
30 - 39	67.6 (66.7 - 68.5)	83.4 (82.7 - 84.1)	79.7 (79.0 - 80.5)	72.2 (71.4 - 73.0)	0.994*	1.20 (1.13 - 1.28)	1.13 (1.04 - 1.22)	1.19 (1.12 - 1.28)	1.04 (0.98 - 1.11)	0.617*
40 - 49	70.7 (69.7 - 71.6)	86.8 (86.1 - 87.5)	85.4 (84.8 - 86.0)	77.84 (77.1 - 78.6)	0.112**	1.30 (1.21 - 1.39)	1.49 (1.37 - 1.63)	1.72 (1.60 - 1.86)	1.26 (1.18 - 1.34)	0.897*
50 - 59	74.1 (72.9 - 75.2)	84.7 (83.8 - 85.7)	85.3 (84.6 - 86.0)	79.0 (78.2 - 79.8)	0.005**	1.54 (1.42 - 1.67)	1.32 (1.20 - 1.46)	1.79 (1.65 - 1.95)	1.32 (1.23 - 1.42)	0.787*
**Household area (%)**										
Urban	48.2 (47.3 - 49.1)	73.8 (73.0 - 74.5)	73.7 (73.0 - 74.4)	68.8 (68.2 - 69.4)	0.094**	Ref				
Rural	75.7 (75.2 - 76.2)	87.3 (86.9 - 87.7)	84.4 (84.0 - 84.8)	79.1 (78.6 - 79.7)	0.988*	2.09 (1.98 - 2.21)	1.54 (1.44 - 1.66)	1.35 (1.27 - 1.43)	1.27 (1.21 - 1.34)	0.039*
**Region (%)**										
Bangkok	40.3 (38.8 - 41.7)	65.8 (64.5 - 67.1)	64.4 (63.0 - 65.7)	59.8 (58.6 - 60.9)	0.157**	Ref				
Central	59.4 (58.3 - 60.4)	79.2 (78.4 - 80.1)	75.9 (75.1 - 76.7)	70.1 (69.4 - 70.9)	0.719*	1.18 (1.08 - 1.28)	1.41 (1.29 - 1.54)	1.50 (1.38 - 1.63)	1.37 (1.28 - 1.47)	0.646*
North	73.6 (72.6 - 74.6)	88.5 (87.8 - 89.3)	87.0 (86.3 - 87.7)	82.4 (81.6 - 83.3)	0.211**	1.88 (1.71 - 2.06)	2.68 (2.40 - 2.99)	3.04 (2.76 - 3.36)	2.65 (2.44 - 2.88)	0.302*
North-east	77.3 (76.5 – 78.0)	87.9 (87.3 - 88.5)	85.2 (84.7 - 85.8)	79.9 (79.2 - 80.7)	0.947*	2.28 (2.09 - 2.48)	2.39 (2.16 - 2.64)	2.67 (2.44 - 2.92)	2.21 (2.05 - 2.39)	0.937*
South	67.9 (66.5 - 69.2)	86.1 (85.1 - 87.1)	84.6 (83.7 - 85.5)	79.6 (78.4 - 80.5)	0.177**	1.46 (1.32 - 1.61)	2.07 (1.83 - 2.33)	2.55 (2.30 - 2.83)	2.14 (1.96 - 2.33)	0.240*
**Marital status (%)**										
Never married	58.2 (57.2 - 59.2)	76.8 (76.0 - 77.7)	75.6 (74.8 - 76.4)	69.1 (68.3 - 69.9)	0.129**	Ref				
Married	70.0 (69.4 - 70.6)	84.9 (84.4 - 85.3)	82.7 (82.3 - 83.1)	76.5 (76.0 - 77.0)	0.210**	0.94 (0.88 - 0.99)	1.08 (1.01 - 1.16)	1.06 (0.99 - 1.12)	1.05 (1.00 - 1.11)	0.724*

Meeting Enhance MVPA Guidelines	Prevalence (95% CI)					Adjusted OR (95% CI)				
	2001	2004	2009	2015	P-value	2001	2004	2009	2015	P-value
Formerly married	63.8 (61.8 - 65.8)	81.0 (79.4 - 82.7)	81.7 (80.4 - 83.1)	74.5 (73.1 - 75.9)	0.021**	0.72 (0.64 - 0.81)	0.89 (0.78 - 1.02)	1.05 (0.94 - 1.18)	0.95 (0.87 - 1.04)	0.399*
**Religion (%)**										
Buddhist	66.4 (66.0 - 66.9)	82.5 (82.1 - 82.9)	80.8 (80.5 - 81.2)	74.1 (73.7 - 74.5)	0.152**	Ref				
non-Buddhist	72.5 (70.5 - 74.6)	85.4 (83.8 - 87.1)	84.0 (82.4 - 85.5)	78.4 (76.7 - 80.1)	0.156**	1.43 (1.27 - 1.60)	1.17 (1.01 - 1.37)	1.29 (1.14 - 1.46)	1.17 (1.04 - 1.31)	0.656*
**Employment status (%)**										
Employed	69.5 (69.0 - 70.0)	86.1 (85.7 - 86.4)	83.6 (83.3 - 84.0)	76.7 (76.3 - 77.1)	0.213**	Ref				
Unemployed	53.8 (52.5 - 55.0)	66.7 (65.6 - 67.8)	67.9 (66.9 - 69.0)	64.0 (63.1 - 65.0)	0.390*	0.59 (0.55 - 0.62)	0.38 (0.36 - 0.41)	0.48 (0.46 - 0.51)	0.57 (0.54 - 0.60)	0.740*
**Highest education level (%)**										
None	74.2 (71.7 - 76.6)	80.1 (77.8 - 82.4)	77.8 (76.0 - 79.6)	70.6 (69.3 - 71.9)	0.391*	Ref				
Primary	75.5 (75.0 - 76.1)	86.4 (86.0 - 86.9)	84.5 (84.0 - 85.0)	80.6 (80.0 - 81.2)	0.816*	1.01 (0.88 - 1.16)	1.36 (1.16 - 1.59)	1.32 (1.18 - 1.48)	1.51 (1.4 - 1.63)	0.288*
Secondary	54.8 (53.8 - 55.8)	78.4 (77.6 - 79.2)	78.3 (77.6 - 79.0)	72.3 (71.6 - 73.1)	0.084**	0.57 (0.49 - 0.65)	1.22 (1.03 - 1.43)	1.25 (1.11 - 1.41)	1.18 (1.09 - 1.27)	0.069**
Higher education	44.1 (42.6 - 45.6)	77.1 (75.9 - 78.3)	76.5 (75.6 - 77.4)	69.0 (68.2 - 69.9)	0.108**	0.39 (0.33 - 0.45)	1.08 (0.91 - 1.28)	1.31 (1.16 - 1.48)	1.21 (1.12 - 1.31)	0.004**
Unspecified	52.6 (41.7 - 63.4)	68.2 (60.0 - 76.4)	80.1 (72.8 - 87.5)	63.3 (57.2 - 69.3)	0.001**	0.58 (0.36 - 0.94)	0.87 (0.57 - 1.34)	1.29 (0.81 - 2.14)	0.82 (0.63 - 1.08)	0.677*

Legend: CI = confidence interval; OR = odds ratio adjusted for all other variables in the table; ref = reference group; * = linear model; ** = quadratic model
